# Influence of Primary Care Physician Availability and Socioeconomic Deprivation on Breast Cancer from 1988 to 2008: A Spatio-Temporal Analysis

**DOI:** 10.1371/journal.pone.0035737

**Published:** 2012-04-20

**Authors:** Lung-Chang Chien, Anjali D. Deshpande, Donna B. Jeffe, Mario Schootman

**Affiliations:** Division of Health Behavior Research, Department of Internal Medicine, Washington University School of Medicine, St. Louis, Missouri, United States of America; Yale University School of Medicine, United States of America

## Abstract

**Background:**

Breast cancer is the most commonly diagnosed cancer and the second leading cause of cancer death among women in the United States. It is unclear how county-level primary care physician (PCP) availability and socioeconomic deprivation affect the spatial and temporal variation of breast cancer incidence and mortality.

**Methods:**

We used the 1988–2008 public-use county-based data from nine Surveillance, Epidemiology, and End Results (SEER) programs to analyze the temporal and spatial disparity of PCP availability and socioeconomic deprivation on early-stage incidence, advanced-stage incidence and breast cancer mortality. The spatio-temporal analysis was implemented by a novel structural additive modeling approach.

**Results:**

Greater PCP availability was significantly associated with higher early-stage incidence, advanced-stage incidence and mortality during the entire study period while socioeconomic deprivation was significantly negatively associated with early-stage incidence, advanced-stage incidence, and mortality up to 1992. However, the observed influence of PCP availability and socioeconomic deprivation varied by county.

**Conclusions:**

We showed important associations of PCP availability and socioeconomic deprivation with the three breast cancer indicators. However, the effect of these associations varied over time and across counties. The association of PCP availability and socioeconomic deprivation was stronger in selected counties.

## Introduction

Breast cancer is the most commonly diagnosed cancer and the second leading cause of cancer death among women in the United States. Breast cancer indicators, such as incidence and mortality, vary over time and across geographic areas in the US [1]. Most of the increase in breast cancer incidence in the 1980's has been attributed to increase in mammography use. A subsequent decline in incidence likely reflected the saturation of screening in the 1990's [2] and a reduction in postmenopausal hormone replacement therapy use in the early 2000s [3]. Breast cancer mortality in the U.S. significantly declined 1.9% per year from 1998 to 2006 due to early detection and increased adjuvant therapy use [4]. Temporal trends in breast cancer mortality also varied by state [5]. Studies also have documented local areas where breast cancer risk was elevated [6]–[10].

Two potential explanations for the temporal change and geographic disparities in breast cancer incidence and mortality are primary care physician (PCP) availability and socioeconomic deprivation at the county level. PCP availability is likely to be an important influence on breast cancer indicators as there is clear evidence that physician recommendation for mammography is a strong predictor of its use [11]–[16]. Previous studies showed that women living in areas with fewer PCPs may be less likely to be screened and have higher mortality rates [17], [18]. Also, county-level socioeconomic deprivation may be related to breast cancer incidence and mortality because areas with greater deprivation may have fewer primary care physicians, limited mammography facilities, and fewer resources for mammography use [19]. Therefore, it becomes important to determine if PCP availability and socioeconomic deprivation can explain observed longitudinal trends and geographic patterns of breast cancer incidence and mortality.

The purpose of this study was to investigate the spatial and temporal variation of breast cancer incidence and mortality due to county-level PCP availability and socioeconomic deprivation using the 1998–2008 county-based data from 200 counties in nine Surveillance, Epidemiology, and End Result (SEER) registries. Specifically, two questions were addressed: (a) whether change in PCP availability and socioeconomic deprivation over time is associated with an ascending or descending trend in breast cancer incidence and mortality from 1988 to 2008, and (b) whether the effect of county-level PCP availability and socioeconomic deprivation on breast cancer incidence and mortality is more pronounced in some counties.

## Methods

### Data source and breast cancer indicators

The 1988–2008 county-based data from nine population-based SEER programs were used to derive three breast cancer indicators (early-stage incidence, advanced-stage incidence and mortality), year of diagnosis and county information. The nine SEER areas, including 5 states (Connecticut, Hawaii, Iowa, New Mexico, and Utah) and 4 cities (Atlanta, Detroit, San Francisco, and Seattle), covered 200 counties with about 9% of the United States population. Women age 40 and older were included in the study if they were diagnosed with a first primary breast cancer (ICD-9 codes: 174, 217, 233) and/or if they died from breast cancer (ICD-10 codes: C50, D05, D24) between 1988 and 2008. Subsequent cancers among women with first primary breast cancer were not included. The study period began in 1988, the first year for which detailed data about lymph node involvement was available in order to use the American Joint Commission on Cancer tumor-node-metastasis (TNM) staging system. Early-stage breast cancer consisted of *in situ* breast cancer and invasive breast cancers that were <2 cm at the time of diagnosis. Advanced-stage breast cancer was defined as TNM stage II and stage III tumors, which include tumors >2 cm and/or have spread to nearby lymph nodes, and TNM stage IV cancers which have spread beyond the breast and lymph nodes to other parts of the body. Breast cancer mortality was determined by death certificates. Women with breast cancer who died from other causes were not included in the breast cancer mortality rate. Because the data we used was a public-use dataset, written consent given by the patients for their diagnosis with breast cancer and personal information was not needed. County-level boundary data were obtained from the U.S. Census Bureau.

This study measured the effects of two county-level determinants including a PCP availability index and a socioeconomic deprivation index. PCP availability was defined as the ratio of the number of PCP per 100,000 women age 40 and older in each county, and data were obtained from the Area Resource File for each of the 200 counties in each year during 1988–2008. Included physicians reported their primary specialty area as general or family practice or reported most of their clinical hours in the practice of obstetrics/gynecology or internal medicine [20].

The socioeconomic deprivation index was constructed from a factor analysis of 46 county-level Census variables from the 1990 and 2000 U.S. Census [21]. We selected the 46 variables for analysis from the 2000 census that were identified from four key studies [22]–[25] and our own conceptualization of socioeconomic deprivation. Eight different domains were considered: education, employment, occupation, housing, poverty, racial/ethnic composition, residential stability, and other. We excluded 23 census variables that measured the inverse of a census variable already included or were very similar constructs. One 6-item common factor emerged: percentage without high school education, percentage unemployed, percentage living in crowded housing (>1 person/room), percentage without a car, percentage without a telephone, and percentage of population below federal poverty rate. Cronbach's alpha was 0.93, and 73.6% of the overall variance was explained by this factor. Because our study data also spanned the 1990 census, we calculated the correlation between the 2000 county index score and the 1990 county index score, computing each index score using the same six census variables. The correlation was 0.881, suggesting that counties with high levels of socioeconomic deprivation in 1990 also had high levels of socioeconomic deprivation in 2000. The correlation between the county-level PCP availability index and the socioeconomic deprivation index was 0.03 (p = 0.14).

### Statistical methodology: structural additive regression model

We examined the spatial distributions in breast cancer incidence and mortality rates and possible nonlinear effects using structural additive regression (STAR) models in order to account for temporal autoregressive correlation and spatial autocorrelation among 200 counties during 1988–2008 [26]. Based on these models, we established a varying-coefficient model (VCM) to investigate the influence of the two determinants on the breast cancer indicators over time and a separate random-effects model (REM) to examine the effect of these determinants on the breast cancer indicators in each of the 200 counties. Each of the two models was fitted for the three breast cancer indicators separately.

Assume 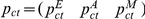 is the county-year-age-race adjusted rate for each breast cancer indicator using the 2000 U.S. standard population, respectively, where *c* ∈(1, 2, …, 200) denotes the index of county, *t* ∈(1, 2, …, 21) denotes the year from 1988 to 2008, *E* denotes early-stage incidence, *A* denotes advanced-stage incidence, and *M* denotes mortality. To implement the time-varying coefficient along with the adjustment of temporal autoregressive and spatial correlation, a VCM can be defined by:
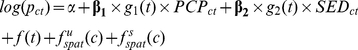
(1)where *α* is an intercept explained as an overall log relative risk for all counties, and 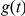 is a second order random walk smoothness prior along with linear predictors. The parameters 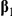 and 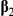 denote temporal fixed effect vectors with dimension 1×21 for the PCP availability variable (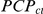) and the socioeconomic deprivation variable (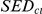), respectively. The function 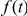 is a time smoother fitted by a penalized spline based on Bayesian P-spline priors [27]. Its functionality is mainly for controlling autoregressive correlations among our longitudinal data. In eq.(1), we used a second order random walk prior to ensure flexibility of the Gaussian errors and diffuse priors for the initial values of the time smoother.

To account for the heterogeneity due to spatial dependence, the spatial effect was decomposed into two terms: an unstructured spatial term 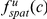 fitted by an exchangeable normal prior 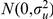 and a structured spatial function 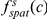 fitted by Markov random fields (MRF). The MRF assumed a conditional autoregressive prior [28] defined as 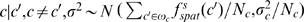. The term *N_c_* is the number of adjacent counties around county *c*, and *c′* ∈*ω_c_* means that county *c′* is one of the neighboring counties *ωc* of county *c*. The two spatial components include a spatially correlated part (structured term) and a spatially uncorrelated part (unstructured term) to distinguish between two types of spatially unobserved covariates, namely, those covariates that examine a strong geographic heterogeneity and those covariates that are identified locally [28], [29]. Two unknown variance components 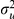 and 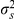 were assigned an inverse Gamma hyper-prior with known hyper-parameters (a, b) = (0.001, 0.001). The intercept was assumed to have a flat prior [30]. This study mainly used the structured spatial effect to show the impact of county location on breast cancer incidence and mortality. The statistical significance of the structured spatial effect relative to the background rate was determined by its 80% posterior probability, with results classified as a statistically significant positive spatial effect, a statistically significant negative spatial effect, or a statistically non-significant spatial effect.

The REM is the same as a generalized linear mixed model with only random effects, but the functionality of the structural additive models makes the spatial heterogeneity estimable along with the estimation of the random effects. It was defined as:
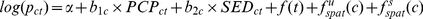
(2)where two random effects, 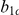 and 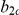, can be explained by the log relative risk for each increment of 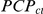 and 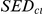 in a specific county *c*. The remaining assumptions of unknown parameters and functions are identical to those used in the VCM. To evaluate the change in the breast cancer indicators attributed to PCP availability and to socioeconomic deprivation, a REM without 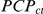, a REM without 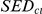 and a REM without both 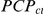 and 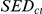 were fitted to compare the results generated from eq.(2), respectively.

All models were fitted using a fully Bayesian influence approach using Markov Chain Monte Carlo techniques, which is carried out by randomly drawing from the full conditional distributions of blocks of parameters conditional on the rest of parameters and the data [29]. More details can be found in Brezger's and Lang's methodological paper [31]. Briefly, for each model, 22,000 iterations were carried out, with the first 2,000 samples used as burn in. We stored every 20th sample from the remaining 20,000 samples, giving a final sample of 1,000 for estimating the model parameters. The significance of the estimates for 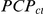 and 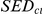 was determined by their 95% CIs. Model diagnostics used the deviance information criterion (DIC) is based on the sum of the posterior mean of the deviance and the effective number of parameters [32]. Maps of the county-level structured spatial function and county-level random effects in VCM and REM displayed the geographic distribution of breast cancer incidence and mortality. The data analysis was implemented by the BayesX 2.01 software package [33].

## Results

### Demographics

From 1988 to 2008, 189,574 women were diagnosed with early-stage breast cancers, 142,338 women were diagnosed with advanced-stage breast cancer, and 57,683 women died of breast cancer (Table 1). The crude rates per 100,000 population of early-stage incidence ranged from 123.5 in New Mexico to 176.4 in Seattle. New Mexico also had the lowest crude rate of advanced-stage incidence with 104.3 per 100,000 population and Detroit had the highest crude rate of advanced-stage incidence and mortality with 126.8 and 58.2 per 100,000 population, respectively. The lowest crude mortality rate was found in Hawaii with 33.2 per 100,000 population.

**Table 1:** Average frequencies and crude rates for three breast cancer indicators in nine SEER areas, 1988–2008.

	# of	Early-stage incidence		Advanced-stage incidence		Mortality	
Area	counties	Frequency	Rate†	Frequency	Rate	Frequency	Rate
San Francisco	5	31,003	164.3	23,054	122.1	9,032	47.9
Connecticut	8	30,138	176.0	20,912	122.1	9,357	54.7
Atlanta	5	16,345	142.9	12,975	113.4	4,787	41.9
Hawaii	5	9,231	166.5	5,920	106.8	1,841	33.2
Iowa	99	22,101	153.7	17,182	119.5	7,777	54.1
Detroit	3	29,867	155.9	24,295	126.8	11,144	58.2
New Mexico	33	9,697	123.5	8,190	104.3	3,324	42.4
Utah	29	9,610	128.3	7,964	106.3	2,963	39.5
Seattle	13	31,582	176.4	21,846	122.0	7,458	41.7
Total	200	189,574	155.9	142,338	119.5	57,683	42.4

† Median of county-year-age-race specific standardized crude rate (per 100,000 women).

### Model diagnostics and spatial variances

Table 2 shows that the two types of models had similar DIC values for the three breast cancer indicators, suggesting that both models fit equally well. Variance components show that for the REM the structured spatial component (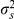) had a larger variance than unstructured spatial component (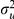) for each breast cancer indicator, suggesting that the spatial heterogeneity played a more dominant role than the random spatial intercepts. The structured spatial variance also was larger than the unstructured spatial variance in the VCM for early-stage and advanced-stage incidence.

**Table 2:** Model diagnostics and variance components of structured and unstructured spatial function in three breast cancer indicators.

	Early-stage incidence		Advanced-stage incidence		Mortality	
	VCM	REM	VCM	REM	VCM	REM
D(θ)	4203.80	4201.45	4200.53	4196.55	4201.14	4200.83
p_d_	216.34	222.74	210.89	213.09	211.87	212.61
DIC	4420.14	4424.19	4411.42	4409.64	4413.01	4413.44
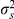 (95% CI)	2.26	3.96	3.18	1.62	0.85	2.16
	(0.76, 4.62)	(1.92, 6.29)	(1.25, 5.64)	(0.43, 3.86)	(0.22, 1.84)	(0.97, 3.94)
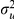 (95% CI)	0.56	0.42	0.43	0.64	0.78	0.52
	(0.02, 1.02)	(0.003, 1.03)	(0.004, 0.94)	(0.09, 1.09)	(0.46, 1.11)	(0.14, 0.96)
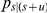	0.80	0.90	0.88	0.72	0.52	0.81

Abbreviation: VCM = varying-coefficient model; REM = random-effects model, D(θ) = posterior mean of the deviance; p_d_ = effect number of parameters; 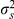 = structured spatial variance; 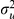 = unstructured spatial variance; 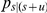 = proportion of the structured spatial variance in total spatial variance.

### Early-stage breast cancer incidence

**Figure 1:**
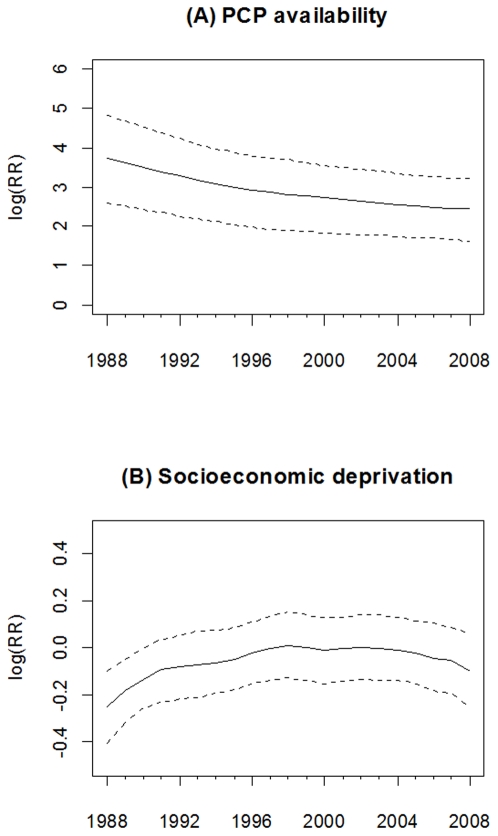
Time-varying estimated coefficient in the VCM for early-stage breast cancer incidence. (A) PCP availability. (B) Socioeconomic deprivation. The dash line is 95% credible interval.

The association (estimated as the log relative risk [logRR]) of PCP availability with early-stage breast cancer incidence declined over time from 3.72 (95% CI = 2.59, 4.81) in 1988 to 2.45 (95% CI = 1.63, 3.22) in 2008, see Figure 1(A). Thus, the lower bound of the 95% CI for each logRR of PCP availability was above zero, suggesting that during this study period higher PCP availability was associated with higher early-stage breast cancer incidence. However, the magnitude of this association declined 34.1% ([3.72–2.45]/3.72) during the 21 year study period. Significant associations between socioeconomic deprivation and early-stage incidence were observed only from 1988 to 1990, where the logRR gradually increased from −0.25 (95% CI = −0.41, −0.10) in 1988 to −0.13 (95% CI = −0.26, −0.001) in 1990, see Figure 1(B).

**Figure 2:**
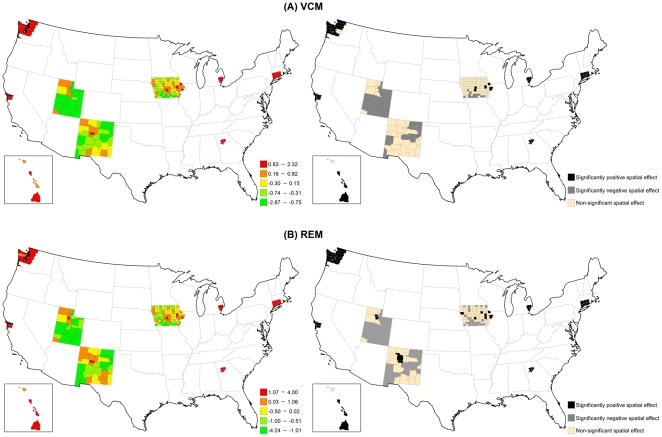
Maps for structured spatial function in the VCM and REM for early-stage breast cancer incidence. (Left) Estimated structured spatial effect, where the range was categorized by 5-quantiles. (Right) 80% posterior probabilities, where black colour means significantly positive spatial effect, grey colour means significantly negative spatial effect, and tan colour means non-significantly spatial effect.

**Figure 3:**
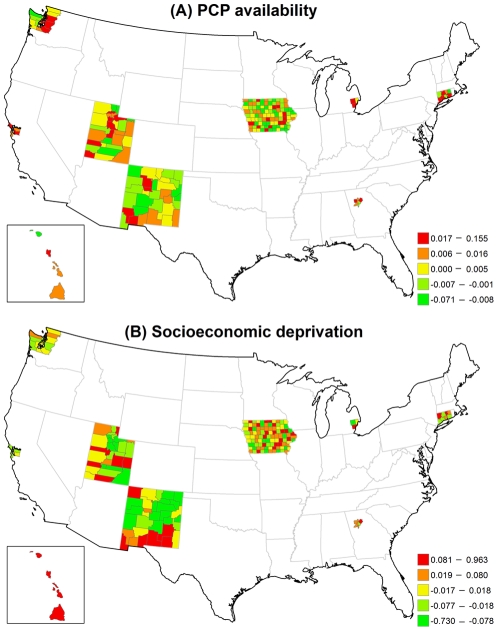
Maps for estimated county-level random effects in the REM for early-stage breast cancer incidence. (A) PCP availability. (B) Socioeconomic deprivation.

Figure 2 displays the structured spatial variability in early-stage incidence across 200 counties for the VCM and REM approaches. Significantly positive spatial effects, determined by 80% posterior probability of logRR>0, was present in 41 counties in the VCM and 48 counties in the REM, mostly in metropolitan SEER areas. This finding suggests that at least 20% of counties had significantly elevated early-stage incidence due to their locations after controlling for county-specific PCP availability and socioeconomic deprivation in either model. Figures 3(A) and 3(B) display the county-specific associations of PCP availability and socioeconomic deprivation with early-stage incidence estimated by the REM for all 200 counties. The association of both determinants with early-stage incidence varied by county, where the variances of random effects were 0.13 (95% CI = 0.002, 0.94) for PCP availability and 0.18 (95% CI = 0.06, 0.35) for socioeconomic deprivation.

The county-level influence of PCP availability and socioeconomic deprivation varied across counties in different SEER areas (Table 3). Larger variances suggest large geographic disparity for the county-specific association between each of the two risk factors and early-stage breast cancer incidence. The association between PCP availability and early-stage breast cancer incidence varied the most across the five counties in Hawaii (variance = 0.0042) and least across the 99 counties in Iowa (variance = 0.0002). Across all counties, the RR ranged from a low of 0.93 to a high of 1.17. In contrast, the association between socioeconomic deprivation and early-stage breast cancer incidence varied the most across the 33 counties in New Mexico (variance = 0.1191) and the least across the 5 counties in the San Francisco area (variance = 0.0014). In one New Mexico county, increasing socioeconomic deprivation was strongly associated with a lower incidence of early-stage breast cancer (RR = 0.48) while in another New Mexico county increasing socioeconomic deprivation increased the early-stage breast cancer incidence (RR = 2.62).

**Table 3:** Geographic disparity of county-specific relative risk of PCP availability and socioeconomic deprivation on three breast cancer indicators by SEER areas, presented by RR.

		Early-stage incidence					
		PCP availability			Socioeconomic deprivation		
Area	N†	Range	Mean	Variance	Range	Mean	Variance
San Francisco	5	1.01–1.08	1.03	0.0008	0.90–1.00	0.96	0.0014
Connecticut	8	0.98–1.06	1.02	0.0006	0.95–1.13	1.02	0.0050
Atlanta	5	0.99–1.08	1.02	0.0013	0.95–1.23	1.08	0.0104
Hawaii	5	0.99–1.15	1.04	0.0042	0.82–1.35	1.14	0.0380
Iowa	99	0.98–1.05	1.00	0.0002	0.80–1.27	1.01	0.0083
Detroit	3	1.00–1.03	1.02	0.0003	0.92–1.13	1.02	0.0110
New Mexico	33	0.93–1.09	1.00	0.0005	0.48–2.62	0.96	0.1191
Utah	29	0.97–1.17	1.01	0.0012	0.71–1.81	1.01	0.0435
Seattle	13	0.99–1.05	1.01	0.0004	0.93–1.20	1.01	0.0044

		Advanced-stage incidence					
		PCP availability			Socioeconomic deprivation		
Area	N	Range	Mean	Variance	Range	Mean	Variance
San Francisco	5	1.13–1.54	1.24	0.0309	0.99–1.01	1.00	0.0002
Connecticut	8	0.96–1.48	1.10	0.0323	0.98–1.03	1.00	0.0004
Atlanta	5	0.96–1.70	1.27	0.0716	1.01–1.07	1.03	0.0005
Hawaii	5	0.98–2.50	1.35	0.4201	0.90–1.07	1.02	0.0046
Iowa	99	0.92–1.58	1.02	0.0060	0.92–1.11	1.00	0.0007
Detroit	3	1.03–1.37	1.24	0.0337	0.98–1.04	1.01	0.0007
New Mexico	33	0.91–2.14	1.04	0.0407	0.84–1.17	0.99	0.0046
Utah	29	0.95–3.29	1.15	0.2000	0.90–1.08	1.00	0.0016
Seattle	13	0.95–1.46	1.15	0.0274	0.95–1.06	1.00	0.0007

		Mortality					
		PCP availability			Socioeconomic deprivation		
Area	N	Range	Mean	Variance	Range	Mean	Variance
San Francisco	5	1.66–3.07	2.24	0.4232	0.98–1.00	0.98	0.0001
Connecticut	8	0.71–3.54	1.63	1.1179	0.96–1.06	1.01	0.0012
Atlanta	5	1.05–3.98	2.59	1.1458	1.02–1.11	1.06	0.0015
Hawaii	5	0.88–11.66	3.46	21.2464	0.69–1.13	1.01	0.0314
Iowa	99	0.82–8.63	1.14	0.6398	0.77–1.22	1.01	0.0037
Detroit	3	2.06–3.11	2.43	0.3451	0.96–1.05	1.00	0.0024
New Mexico	33	0.85–18.03	1.61	8.7430	0.77–1.22	0.98	0.0126
Utah	29	0.85–22.60	2.25	18.3064	0.79–1.25	0.99	0.0094
Seattle	13	0.80–3.96	1.65	0.8257	0.93–1.23	1.01	0.0060

† N = number of counties; PCP = primary care physicians.

### Advanced-stage breast cancer incidence

**Figure 4:**
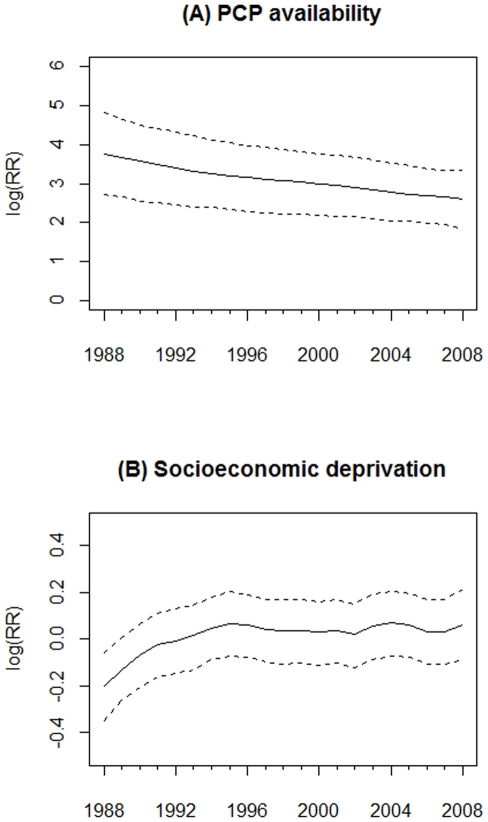
Time-varying estimated coefficient in the VCM for advanced-stage breast cancer incidence. (A) PCP availability. (B) Socioeconomic deprivation. The dash line is 95% credible interval.

Figure 4(A) shows a strong association between PCP availability and advanced-stage incidence over time since the 95% CI of the time-varying logRR excluded zero in each year. The association declined slightly from 1988 to 2008. Figure 4(B) shows that the socioeconomic deprivation and advanced-stage incidence were only statistically associated in 1988 (logRR = −0.20; 95% CI = −0.35, −0.06), but not after 1988.

**Figure 5:**
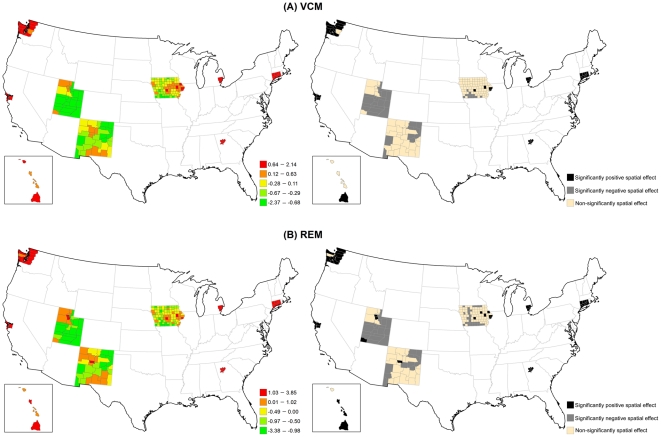
Maps for structured spatial function in the VCM and REM for advanced-stage breast cancer incidence. (Left) Estimated structured spatial effect, where the range was categorized by 5-quantiles. (Right) 80% posterior probabilities, where black colour means significantly positive spatial effect, grey colour means significantly negative spatial effect, and tan colour means non-significantly spatial effect.

**Figure 6:**
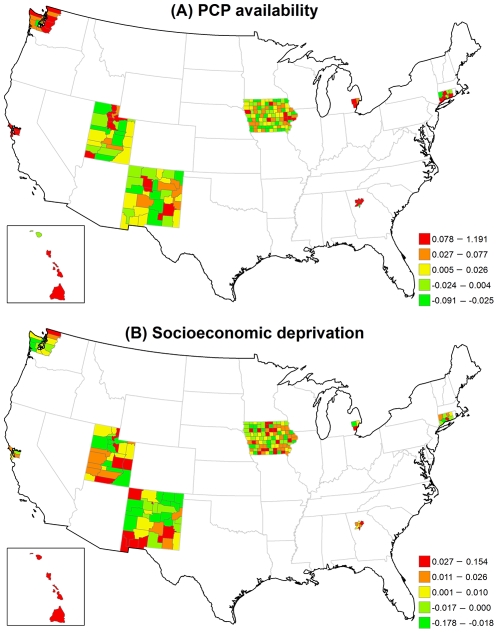
Maps for estimated county-level random effects in the REM for advanced-stage breast cancer incidence. (A) PCP availability. (B) Socioeconomic deprivation.

Figure 5 illustrates that the structured spatial effect in advanced-stage breast cancer incidence varied across the counties based on the VCM and REM. Among 200 counties, 37 counties in the VCM and 45 counties in the REM had significantly positive spatial estimates. Most of these counties were located in metropolitan SEER areas. Figure 6 indicates that the county-specific logRR varied across counties, where the variances of random effects were 1.22 (95% CI = 0.002, 6.81) for PCP availability and 0.04 (95% CI = 0.001, 0.14) for socioeconomic deprivation.

Table 3 shows that the variance of the association of PCP availability with advanced-stage breast cancer incidence across the counties for each of the SEER areas was generally larger than the variance of the association of socioeconomic deprivation with advanced-stage breast cancer incidence. Hawaii and Utah had the largest variances, indicating that the association between PCP availability and advanced-stage incidence varied the most across counties in these two areas.

### Breast cancer mortality

**Figure 7:**
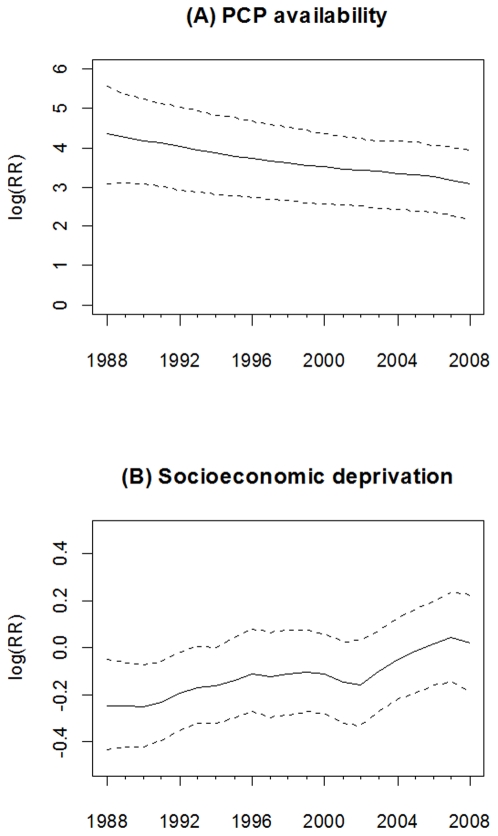
Time-varying estimated coefficient in the VCM for breast cancer mortality. (A) PCP availability. (B) Socioeconomic deprivation. The dash line is 95% credible interval.

As shown in Figure 7(A), PCP availability had a consistently positive and significant association with breast cancer mortality from 1988 to 2008. The logRR of PCP availability declined from 4.35 (95% CI = 3.08, 5.58) in 1988 to 3.08 (95% CI = 2.16, 3.94) in 2008. As shown in Figure 7(B), only during 1988–1992 was socioeconomic deprivation significantly associated with breast cancer mortality, whereby counties with higher socioeconomic deprivation had lower logRR of breast cancer mortality.

**Figure 8:**
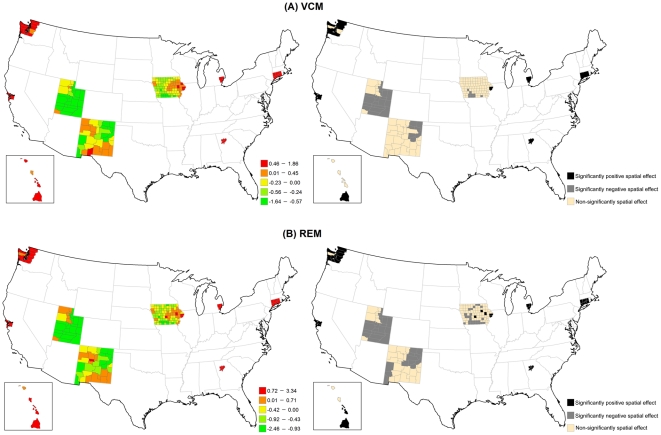
Maps for structured spatial function in the VCM and REM for breast cancer mortality. (Left) Estimated structured spatial effect, where the range was categorized by 5-quantiles. (Right) 80% posterior probabilities, where black colour means significantly positive spatial effect, grey colour means significantly negative spatial effect, and tan colour means non-significantly spatial effect.

**Figure 9:**
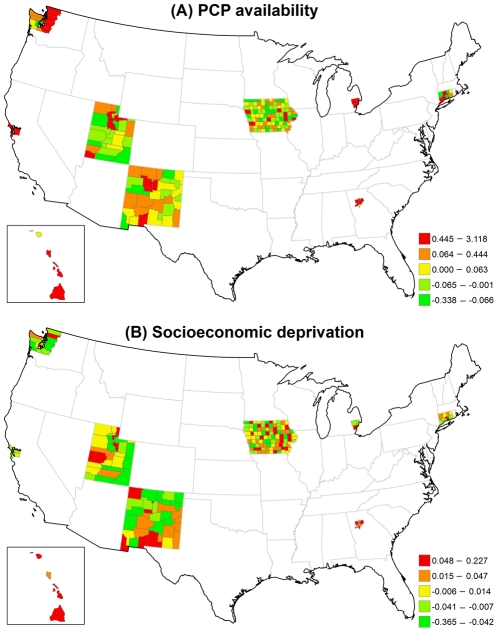
Maps for estimated county-level random effects in the REM for breast cancer mortality. (A) PCP availability. (B) Socioeconomic deprivation.

Figure 8 shows the large structured geographic variability in breast cancer mortality across the 200 SEER counties in the VCM and REM, especially in metropolitan SEER areas. Of the 2000 SEER counties, 34 counties in the VCM (17.0%) and 39 counties in the REM (19.5%) had at least an 80% posterior probability of increased breast cancer mortality (logRR>0). Figure 9 shows that county-specific effects of PCP availability and socioeconomic deprivation varied across counties based on the REM, where the estimated variances of the random effects were 5.76 (95% CI = 0.01, 22.00) for PCP availability and 0.10 (95% CI = 0.004, 0.32) for socioeconomic deprivation.

The county-specific association of PCP availability with the mortality rate showed greater variability than the county-specific association of socioeconomic deprivation with the mortality rate in each SEER area (Table 3). The largest variance of the association between PCP availability and the mortality rate was in Hawaii, although the variability in this association was also large across counties in New Mexico and Utah. For many counties, increasing PCP availability was associated with an increasing mortality rate.

## Discussion

The purpose of this study was to examine the association and geographic disparity of PCP availability and socioeconomic deprivation over time with three breast cancer indicators using spatio-temporal analysis with structural additive models of county-level SEER data from 1988–2008. Our study showed two main findings. First, increasing PCP availability was significantly associated with increasing early-stage breast cancer incidence, advanced-stage incidence, and mortality over the entire study period, but socioeconomic deprivation was only significantly associated with the three breast cancer indicators during the early years of the study period. Second, large geographic disparities across the SEER counties were observed in the associations of PCP availability and socioeconomic deprivation with each of the three breast cancer indicators.

As described, PCP availability increased the likelihood of early-stage breast cancer incidence, but the association declined over time. A likely explanation is the increased prevalence of mammography service and use since the late 1980s. This result is consistent with a previous study of the benefit of screening mammography on early-stage breast cancer diagnoses, which found overall age-adjusted breast cancer incidence rates increased 25% from the early 1980s to 1993, and then dropped by 18% in 2004 [34]. A positive relationship between PCP availability and early-stage breast cancer incidence also has been reported in other studies [18], [35], and we further illustrated its change over time in Figure 1. Moreover, we also found a positive association of PCP availability with breast cancer mortality over time. Although some studies showed that higher PCP availability was associated with lower mortality for some health conditions [36]–[38], we observed a positive association, which might have been the result of confounding by rurality, whereby counties considered to be more urban had higher PCP availability and higher mortality (see Figure 8). The time-varying socioeconomic deprivation was significantly negatively associated with early-stage incidence from 1988 to 1990 in our study. Higher socioeconomic deprivation may have led to lower screening mammography use resulting in lower early-stage breast cancer rates [39]. Targeted efforts nationally to increase screening among women living in areas with high socioeconomic deprivation might very well account for the lack of significant associations between socioeconomic deprivation and early-stage incidence after 1990.

Our second finding showed large geographic disparities across the SEER counties in the association of PCP availability and socioeconomic deprivation with each breast cancer indicator; in some counties there were positive associations and in other counties there were negative associations. The geographic disparity of PCP availability can be explained by Medicare beneficiaries only residing in some counties with higher levels of PCP availability, which have fewer preventable hospitalizations and lower death rates [40]. The posterior probability of the structured spatial effect in the VCM and REM identified specific counties where significant associations between each of PCP availability and socioeconomic deprivation and all three breast cancer indicators were observed (Figures 2, 5 & 8). The positive associations between each of PCP availability and socioeconomic deprivation and the two early- and advanced-stage breast cancer incidence indicators were significant for at least 20% of the 200 SEER counties, but the associations between each of PCP availability and socioeconomic deprivation and breast cancer mortality was significant for less than 20% of these SEER counties. This finding suggests that the spatial function could identify more significantly elevated incidence than mortality for breast cancer in these 200 SEER counties. The observed significance of spatial effects in the study areas confirms that spatial heterogeneity should not be ignored, and was most pronounced in metropolitan SEER areas. More importantly, these results can be used to target breast cancer detection programs or prevention and control activities to counties with elevated breast cancer incidence and mortality rates and showed the importance of the local influence of PCP availability and socioeconomic deprivation on breast cancer indicators.

A major strength of our study was the sophistication of the statistical modeling strategy. The STAR model goes beyond previously used methods because it provides flexible functions to perform the time- and space-varying influence of determinants on the breast cancer indicators of interest. This modeling approach could be used in future investigation of spatio-temporal variations in risk factors for other health conditions. In addition, the STAR model takes into account data from neighboring counties, so our results are less affected by small numbers than traditional frequentist approaches.

Our study also included two limitations. First, our results may be affected by scattered areas with a lack of neighboring counties in metropolitan SEER areas, such as Atlanta, Detroit, Seattle, San Francisco, and Connecticut. Ignoring these neighboring counties that were not part of the SEER program data may have affected the results of models. The influence of the neighboring counties on counties near the outside boundary of each SEER area may have been underestimated because those neighboring counties were ignored. Second, the STAR model does not support the implementation of space-time interaction, which resulted in our inability to examine geographic variation over time, but this was not a purpose of our study.

To sum up, this study showed important influences of PCP availability and socioeconomic deprivation on three breast cancer indicators in both temporal trends and geographic disparities. The time-varying association of PCP availability was stronger than that of socioeconomic deprivation. The SEER area-specific PCP availability also displayed larger geographic disparities than socioeconomic deprivation, especially in advanced-stage incidence and mortality.
